# Contaminant Removal from Nature’s Self-Cleaning Surfaces

**DOI:** 10.1021/acs.nanolett.3c00257

**Published:** 2023-05-08

**Authors:** Sreehari Perumanath, Rohit Pillai, Matthew K. Borg

**Affiliations:** † Mathematics Institute, University of Warwick, Coventry CV4 7AL, U.K.; ‡ School of Engineering, University of Edinburgh, Edinburgh EH9 3FB, U.K.

**Keywords:** Cicadas, self-cleaning, droplets, nanoparticles, superhydrophobic surfaces

## Abstract

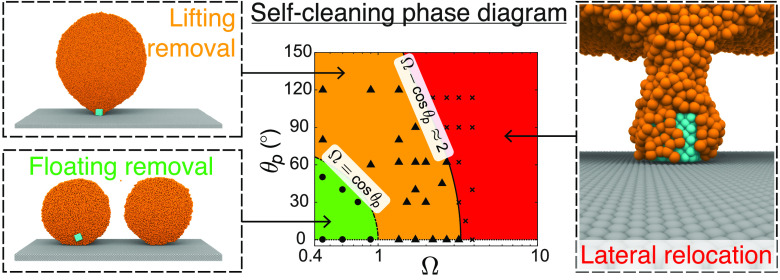

Many organisms in nature have evolved superhydrophobic surfaces that leverage water droplets to clean themselves. While this ubiquitous self-cleaning process has substantial industrial promise, experiments have so far been unable to comprehend the underlying physics. With the aid of molecular simulations, here we rationalize and theoretically explain self-cleaning mechanisms by resolving the complex interplay between particle–droplet and particle–surface interactions, which originate at the nanoscale. We present a universal phase diagram that consolidates (a) observations from previous surface self-cleaning experiments conducted at micro-to-millimeter length scales and (b) our nanoscale particle–droplet simulations. Counterintuitively, our analysis shows that an upper limit for the radius of the droplet exists to remove contaminants of a particular size. We are now able to predict when and how particles of varying scale (from nano-to-micrometer) and adhesive strengths are removed from superhydrophobic surfaces.

Nature has evolved diverse biological surfaces that self-clean by passively removing any contaminants that build up on them. Exemplars include insects (cicadas and planthoppers), reptiles (geckos), and plants (lotus).^1−6^ The common thread connecting these biodiverse organisms is that they all have water-repellent, or *superhydrophobic*, wax-coated surfaces,^2,7^ on which dew condenses to form near-spherical droplets at nucleation sites. The formation of droplets enables self-cleaning in two distinct modes. In the first mode of self-cleaning (see Figure 1(a)), when neighboring condensate droplets coalesce on superhydrophobic surfaces, a portion of the excess surface energy released is converted into translational kinetic energy and the merged droplet jumps away from the surface.^8−10^ This “coalescence-induced jumping” of water droplets is observed across a wide range of length scales, from nanometer-sized^10−12^ to millimeter-sized^13^ droplets, and is the primary mechanism used by cicadas for removing *individual contaminants* from their wings.^2^ The second mode of self-cleaning occurs once the condensing droplets’ radii *R* become comparable to the capillary length of the liquid , where droplets can roll-off the surface picking up *many contaminants* at a time,^14−17^ along the surface of the droplet (see Figure 1(b)). Here, γ is the liquid–vapor interfacial tension, ρ is the liquid’s density, and *g* is the acceleration due to gravity. This second mode of droplet rolling, commonly observed on lotus leaves, is called *the lotus effect*. Notably, in contrast to jumping droplets, the lotus effect only gains importance for millimeter-sized water droplets, since the capillary length for water is *l*_c_ ∼ 10^–3^ m. These observations of droplet-induced cleaning on nature’s surfaces have inspired researchers to develop similar functioning surfaces^18−20^ for solar panels,^21^ automobile surfaces, and wind shields,^22,23^ however, with limited technological advances.

**Figure 1:**
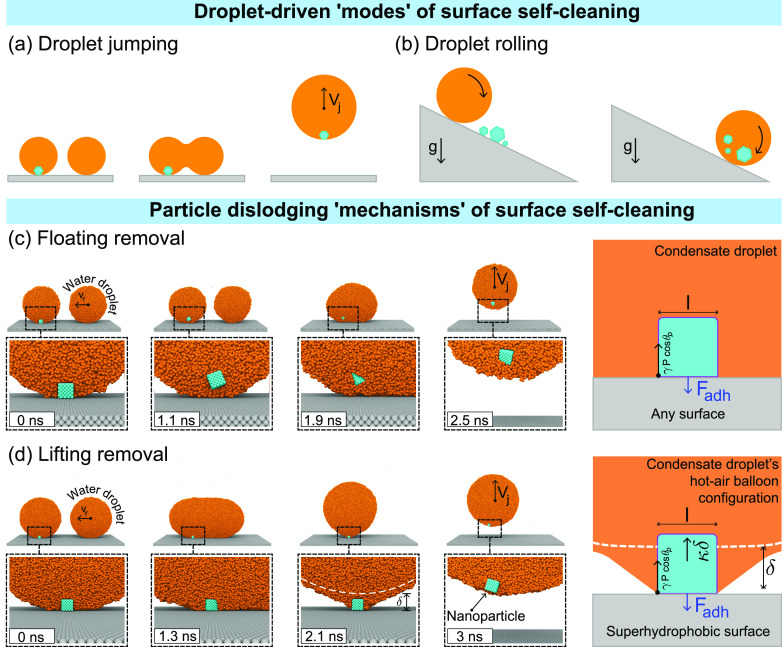
Different *modes* of surface self-cleaning: (a) coalescence-induced droplet jumping and (b) droplet rolling on superhydrophobic surfaces. Different particle removal *mechanisms* of surface self-cleaning utilized by the jumping mode in our MD simulations: (c) Floating removal of a nanoparticle from a superhydrophobic surface (*R* = 7.2 nm, θ_p_ = 0°, Ω = 0.45). Note that the underlying surface does not need to be superhydrophobic for floating removal to occur. Here, the particle gets dislodged from the surface prior to droplet coalescence due to high attraction from the liquid molecules overcoming the surface adhesive forces. The last panel shows the dominant forces acting on the nanoparticle when it is about to undergo floating removal. (d) Lifting removal of a nanoparticle from a superhydrophobic surface (*R* = 15.1 nm, θ_p_ = 62°, Ω = 1.71). Note that the underlying wall must be superhydrophobic for lifting removal to occur. Here, the condensate droplet–nanoparticle system is observed to attain a hot-air balloon configuration toward the end of coalescence as shown in the third panel before picking up the nanoparticle from the surface. The last panel shows the dominant forces acting on the nanoparticle while the system is in the hot-air balloon configuration during lifting removal. The white dashed line indicates a hypothetical undisturbed droplet profile. In (c, d), the frontal halves of both spherical droplets are not shown for better visualization of the nanoparticle.

Despite providing some insight into the self-cleaning processes found in nature, current experiments have limitations in accessing the required small length and time scales as well as the complex interplay of surface chemistry and nanoscale fluid dynamics involved in these processes. Previous experiments have tried to explain how rolling^6,15−17^ and jumping droplets^2,4,5^ remove contaminants from low-wetting surfaces. Intuitively, one would expect hydrophilic contaminants to be preferentially removed by water droplets given the stronger attractive force between the contaminant and the water droplet. However, anomalously, this is not generally observed and its underlying reason is not completely understood. For example, on superhydrophobic surfaces, as demonstrated by Geyer et al.,^6^ small hydrophilic particles are not removed by rolling droplets, while hydrophobic ones are. Additionally, the time scale of coalescence for experimentally studied droplets with *R* > 100 μm is of the order of 100 μs, and thus, it is challenging to experimentally investigate various stages of the particle removal processes that span over much smaller time scales.

Here, we propose a new theoretical model that rationalizes the removal of particles in two fundamental self-cleaning mechanisms using droplets that were observed in recent experiments:^2^ (i) *floating removal* (see Figure 1(c), where the underlying wall is superhydrophobic), which we show for the first time can also occur on non-superhydrophobic surfaces, and (ii) *lifting removal* (see Figure 1(d)), which only materializes on superhydrophobic surfaces. Our molecular simulations reveal the origin of these two self-cleaning mechanisms in nature, and our theory shows how to classify them. The foundation of our proposed theory is the derivation of fundamental non-dimensional relationships between particle–droplet and particle–surface interactions, which are common in both modes of self-cleaning surfaces (lotus leaves and cicadas wings), identifying the limiting conditions for floating removal, lifting removal, and no removal. We show that our model is accurate from the nanoscale to the macroscale, and we present our theory on a phase diagram that unifies observations for both: (a) previous surface self-cleaning experiments conducted at micro-to-millimeter length scales and (b) our nanoscale simulations of droplet jumping from contaminated superhydrophobic surfaces studied using molecular dynamics (MD). Additionally, the results also reveal that droplets jumping in a direction normal to the surface that do not remove the particle from it can laterally displace nanoparticles before taking off, which can be used to relocate them instead. This work gives a comprehensive picture of fundamental mechanisms of self-cleaning surfaces, and the proposed theory enables us to specify exactly how to remove or relocate particles using condensate droplets, once their nature and size are known.

Two water nanodroplets are set up in a fully periodic domain in MD at 300 K on a superhydrophobic surface. A metallic nanoparticle of fixed size and shape is placed under one of the droplets to replicate realistic scenarios of dropwise condensation near nucleation sites (see Figure 1(c,d)). The adhesive force between the nanoparticle and the surface is quantified by *F*_adh_ = *p*_d_*l*^2^, where *l* is the length scale of the particle and *p*_d_ is the disjoining pressure. Following equilibration, an impact speed *v*_r_ is given to the droplet without the nanoparticle, with a magnitude smaller than the inertial-capillary velocity  of the droplet, such that it does not affect the coalescence dynamics. Post coalescence, the merged droplet would jump off the superhydrophobic surface with a speed *V*_j_. Further details of the MD simulations are given in the Supporting Information.

From a theoretical standpoint, the removal mechanisms of small-scale particles are driven by a competition between capillary and adhesive forces. Condensate droplets remove particles from a surface if they are able to exert a dislodging force *F*_⊥_ that is normal to the surface, such that *F*_⊥_ > *F*_adh_. For nanoparticle removal, the process is predominantly capillary-driven and the coalescence dynamics has minimal influence over it. That is, the internal flow field of the droplet caused by its coalescence with a neighbor droplet does not assist the detachment of the contaminant. This is evident from our MD simulations in Figure 1(c), where the particle is dislodged well before coalescence begins, and from Figure 1(d), where the particle still adheres to the surface even after the coalescence is complete. As shown in Figure 1(c,d), two types of nanoparticle removal mechanisms are observed:

1. *Floating removal* happens on *any* surface when a droplet fully immerses the nanoparticle, where the attractive force on the nanoparticle from the surrounding liquid molecules, *F*_att_, is large enough to overcome *F*_adh_ (see the last panel of Figure 1(c)). Therefore, we propose that the condition for floating removal is
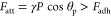
1Here, *F*_att_, which is present throughout the condensation process, acts along the perimeter *P* surrounding the wetted area of the nanoparticle surface and θ_p_ is its equilibrium contact angle with the condensate liquid.^24,25^ In dimensionless form, eq 1 becomes
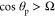
2where Ω ≡ *F*_adh_/*γP* is the adhesive force between the nanoparticle and the underlying surface normalized with the surface tension force between the particle and the condensate liquid. The parameters, Ω and θ_p_, are usually accessible from experiments.
2. *Lifting removal* happens only on a superhydrophobic surface when two neighboring droplets merge and jump after coalescence. Toward the end of coalescence, when the merged droplet is about to jump off the surface with a particle underneath, the droplet surface deforms and we observe it acquires a *hot-air balloon* configuration (see the third panel of Figure 1(d)). Here, in addition to *F*_att_, a supplementary dislodging force *F*_γ_ due to the tension from the deformed droplet interface on a superhydrophobic substrate also appears. This can be modeled by *F*_γ_ = *κδ*, where κ is the stiffness of the liquid surface acting as a linear spring due to its surface tension and δ ≪ *R* is the displacement of the nanoparticle from an “undisturbed” droplet profile (white dashed lines in Figure 1(d)). As detailed in the last panel of Figure 1(d), we propose that the condition for lifting removal is
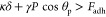
3In non-dimensional form, this becomes
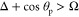
4where Δ ≡ *κδ*/*γP* is a dimensionless parameter that quantifies the additional non-equilibrium pinning force experienced by the nanoparticle. The parameter Δ is obtained via curve fitting from the results of our MD simulations, as the droplet surface displacement, δ, cannot be easily measured in experiments.

It is worth noting that in both modes of self-cleaning (i.e., droplet jumping and rolling) the droplet profile needs to deform to generate a lifting force *F*_γ_. In the droplet rolling mode, however, this lifting force is not perpendicular to the surface,^6^ as it is in the droplet jumping mode (see Figure 1(d)).

The above two conditions represent limits within which each type of nanoparticle removal mechanism can be observed. In Figure 2(a,b), we compare our theoretical predictions with results of our MD simulations and recent experiments, respectively, and observe excellent agreement. The characteristics of this new self-cleaning phase diagram can be summarized as follows:

- Floating removal occurs in the green-colored region, which satisfies the condition (2), where particles spontaneously get immersed in the droplets. Here, the contaminant detachment occurs without any deformation to the enclosing droplet, and thus, droplet jumping or rolling is not necessary for its detachment. As the dislodging outcome is driven mostly by the wettability of the particle and its adhesion with the wall, the underlying wall does not need to be superhydrophobic. We test this hypothesis in Section 2 of the Supporting Information, where we observe floating removal on top of a hydrophilic wall. However, after its detachment, the contaminant stays immersed in the sessile droplet until a secondary mechanism removes the contaminant-laden droplet from the wall. On self-cleaning low-adhesion surfaces, this may be achieved by coalescence-induced jumping of two (see Figure 1(c)) or more droplets,^26^ by droplet rolling,^6^ or by impact coalescence.^27^ MD results validating this mechanism are shown as solid black circles in Figure 2(a). Experiments that observed floating removal on superhydrophobic surfaces with different particle–liquid combinations are shown as hollow black shapes in Figure 2(b).
- Lifting removal can occur in the orange- and green-shaded regions that satisfy condition (4). However, it is most likely to happen in the orange-shaded region only, since floating removal is observed to occur faster in the green-shaded region. Our MD cases validating this mechanism are shown as solid triangles in Figure 2(a), and experimental results are shown as solid black shapes in Figure 2(b). Out of these experiments, those by Wisdom et al. (2013),^2^ Watson et al. (2015),^4^ and Watson et al. (2014)^5^ investigated the droplet jumping mode of self-cleaning and the rest looked into the droplet rolling mode. However, previous works did not uncover the underlying mechanisms of the self-cleaning process. Note that, for the lifting removal mechanism to occur, the radii of the droplets (*R*) must lie between two limits *R*_min_ and *R*_max_ set by the thermophysical properties of the liquid and the length scale of the particle, which is discussed later.
- Inside the red-shaded region, where neither condition (2) nor condition (4) is satisfied, *F*_⊥_ is insufficient to overcome *F*_adh_ irrespective of the value of *R*. Here, nanoparticle removal cannot happen. In such cases, the contact line will either depin from the nanoparticle while the system is in the hot-air balloon configuration (see the third and fourth panels of Figure 3(a)) or, when the contact line is strongly pinned on the nanoparticle, a liquid column forms above it, which is susceptible to the Rayleigh–Plateau (RP) instability (see Figure 3(b)). Both of these scenarios result in the liquid droplet jumping off the surface and leaving the nanoparticle behind.
- As shown in Figure 2(b), our theory captures results of experimental studies on self-cleaning surfaces at micro/millimeter scales too. This includes both modes of self-cleaning: by jumping condensates^2,4,5^ as well as by rolling droplets.^6,15−17^ The details of these experiments are summarized in Section 3 of the Supporting Information. We are now able to explain why, depending on their size, certain hydrophilic contaminants (e.g., silica and calcite particles, indicated by hollow data points with relatively lower θ_p_) undergo floating removal and other less hydrophilic particles (e.g., those composed of poly(methyl methacrylate) and hydrophobized silica, indicated by filled data points with relatively higher θ_p_) undergo lifting removal in experiments. It must be noted that conditions (2) and (4) represent the theoretical limits within which respective particle removal can be seen. In Figure 2(b), although located near the theoretical boundary, some experiments reveal lifting removal in the green-shaded region. While still theoretically possible, this likely arises from using approximate theoretical expressions to estimate *F*_adh_ in previous studies.

**Figure 2:**
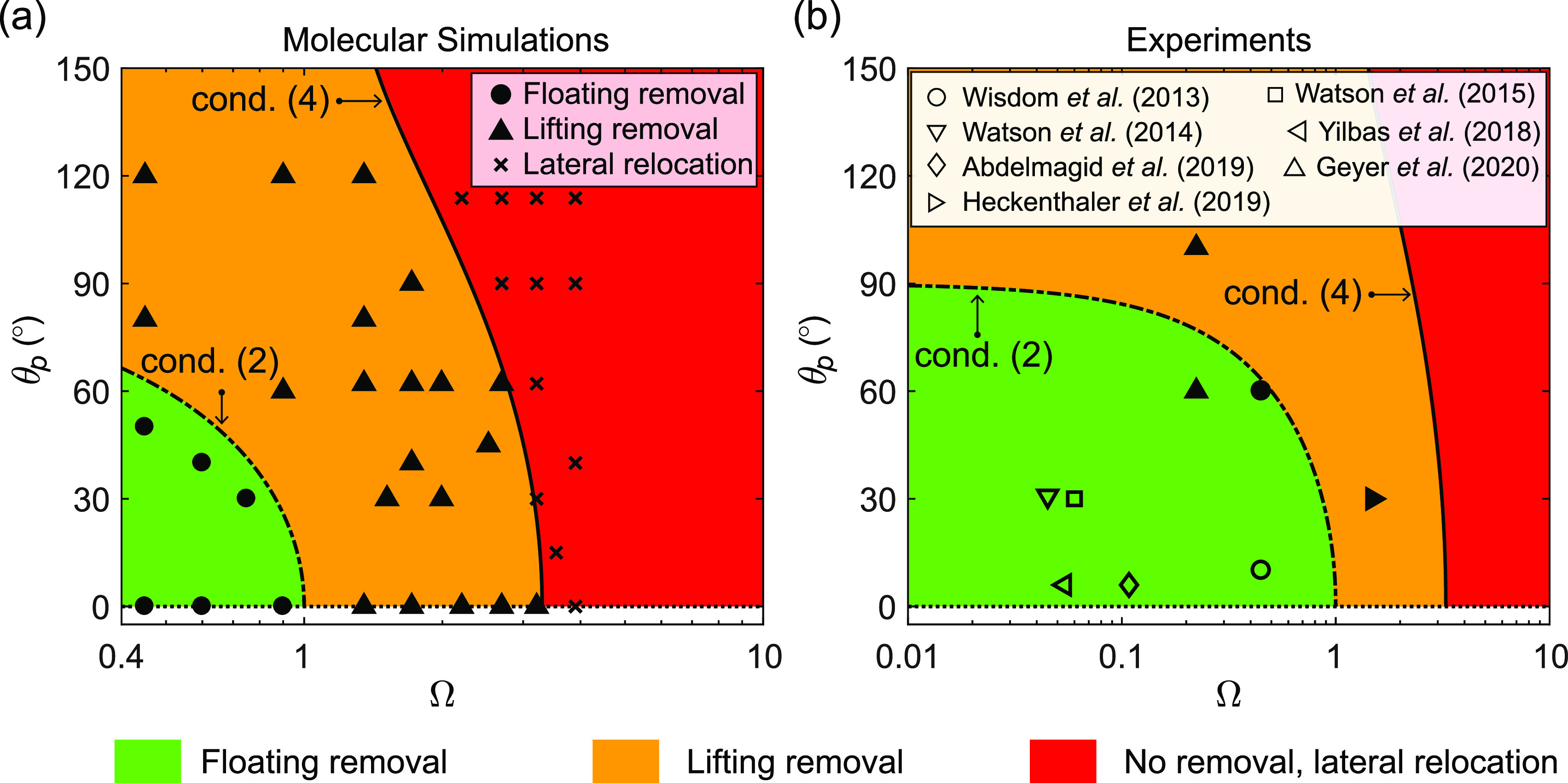
Phase diagram showing nanoparticle removal/relocation on surfaces obtained from (a) our MD simulations and (b) previous experiments, and their comparison with our theoretical predictions. Inside the green-shaded region that is bounded by condition (2), a higher attractive force from the condensate liquid relative to the adhesive force results in floating removal. Lifting removal is observed in the orange-shaded region. Note that such a self-cleaning phase diagram does not give information about the crucial effect the size of the condensate droplet has on lifting removal (see Figure 4). No nanoparticle removal is observed inside the red-shaded region, but they are observed to be transported laterally during coalescence. From (a), we obtained Δ ≈ 2 such that the solid black line separates the lifting removal and lateral relocation regimes. In (b), different symbols correspond to different experimental studies, where we have used Δ = 2 as before. Empty data points represent floating removal, and filled data points indicate self-cleaning either by lifting removal due to jumping droplets or by the lotus effect due to rolling droplets.

**Figure 3:**
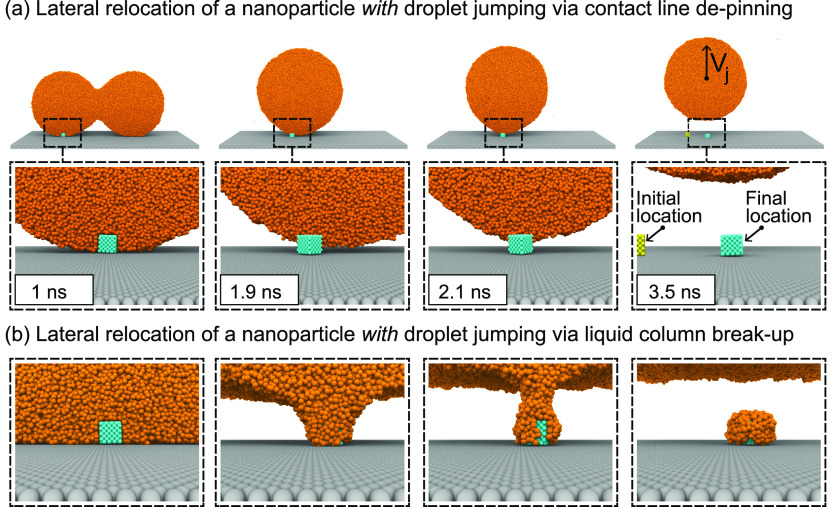
Lateral relocation of nanoparticles on a superhydrophobic surface *with* droplet jumping when the system is in the red-shaded region of the self-cleaning phase diagram. Here, the coalescence of two droplets will drive the nanoparticle to the middle. (a) Droplet jumping after contact line depinning (Ω = 2.7, θ_p_ = 90°). In the fourth image, the initial location of the nanoparticle is shown in yellow color. (b) If the contact line is strongly pinned on the nanoparticle, a liquid column forms and breaks up as the main liquid body tries to leave the surface (Ω = 3.9, θ_p_ = 0°). This replicates a scenario where a large droplet jumps from a superhydrophobic surface trying to remove a relatively small particle. In all the figures, the frontal halves of the liquid bodies are not shown.

To plot condition (4) in Figure 2(a,b), we determine Δ from curve fitting. Presently, there is no theoretical explanation as to why the Δ ≈ 2 (solid black) line separates the lifting and lateral relocation regimes obtained from MD in the self-cleaning phase diagram. However, it is interesting to note that, with this value of Δ, we get δ ≈ *πl*, which corresponds to the critical wavelength for the RP instability-induced breakup of a liquid cylinder with diameter *l*.^28^Figure 3(b) shows such a scenario, where we expect only lateral relocation to occur with droplet jumping (also see Figure S3 in the Supporting Information).

## Effect of Droplet Size

From our MD simulations, we observe that the self-cleaning processes are affected by the size of the enclosing droplet. This means, either of conditions (2) and (4) being satisfied is not enough to ensure self-cleaning from a surface. To this end, we derive theoretical estimates of droplet sizes that are capable of self-cleaning superhydrophobic surfaces. This will tell us the following: (a) which liquid to use for nanoparticle removal and (b) how long dropwise condensation has to occur (where the droplet size gradually increases) to reach the target droplet size under specified ambient conditions.

For floating removal, the nanoparticle should be enclosed in a droplet that can accommodate it once dislodged. This indicates that the droplet diameter needs only to be larger than the length scale of the particle, i.e., 2*R*_min_ > *l*, while no maximum droplet size *R*_max_ needs to exist.

**Figure 4:**
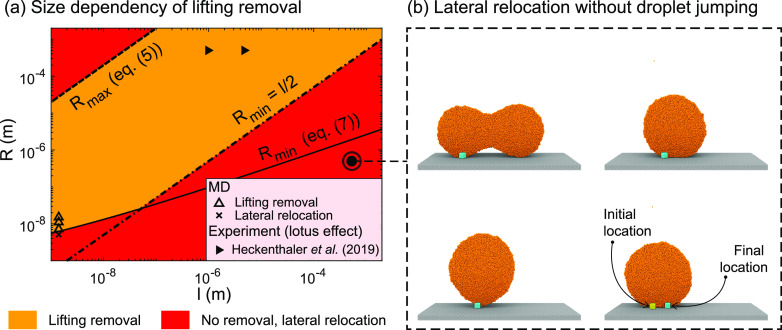
Effect of water droplet size on self-cleaning. In (a), the orange region already satisfies condition (4). The data points and lines are plotted with Ω = 1.32 and θ_p_ = 30°, which correspond to the experiment results of Heckenthaler et al.^7^ Experimental results for the lotus effect are plotted here, because they self-clean using a mechanism which is simlar to that of lifting removal.^6^ In the red-shaded region above *R*_max_, we expect the merged droplet to jump off the surface following the breakup of a liquid column or, in the case of the lotus effect, the rolling droplet would leave the particles behind due to the same breakup of a liquid column. In the red-shaded region below *R*_min_, as shown in (b), droplet coalescence results in the nanoparticle’s lateral relocation but *without* droplet jumping (× data point).

For lifting removal, an upper limit for *R* (*R*_max_) must exist when self-cleaning. For a given particle size *l*, a larger droplet has a smaller stiffness κ, and could therefore have very high values of displacement δ during the hot-air balloon configuration. In this case, a liquid column may form above the nanoparticle that subsequently breaks up due to the RP instability, leaving the nanoparticle behind (similar to the case shown in Figure 3(b)). Importantly, it must be noted that this occurs even though the case may lie in the orange-shaded region of the self-cleaning phase diagram in Figure 2(a,b), which would have ordinarily predicted lifting removal. As such, this additional constraint of *R*_max_ for lifting removal can be estimated by substituting the analytical expression for κ in condition (4), which gives (see Section 2 of the Supporting Information):
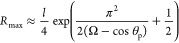
5Figure 4(a) shows the effect of water droplet size on a nanoparticle’s lifting removal or relocation. For Ω = 1.5 and θ_p_ = 30°, corresponding to both MD and experimental data points in the figure, our analysis suggests that the radii of the two coalescing droplets must be smaller than the dashed black line for lifting removal to occur. This may offer an explanation to the anomaly mentioned before in the experiment of Geyer et al.,^6^ where small hydrophobic particles (*l* = 80 nm) were seen to be preferably removed from superhydrophobic surfaces by water droplets but not hydrophilic ones. It must be mentioned that, as we had to estimate Ω in Geyer’s experiments,^6^ this produces uncertainties in the data points in Figure 2(b) as well as building an accurate Figure 4(a) for their experiments. However, using this estimate, we find from eq 5 that *R*_max_ is smaller than the 1 mm droplets used in those experiments, which tells us the droplets may have been too large to remove these small hydrophilic contaminants. For hydrophobic particles, however, *R*_max_ is larger than 1 mm, indicating these cases now lie in the orange/lifting regime of the corresponding size-dependency graph. Care is needed when interpreting self-cleaning results of different particle–liquid wettability, since changes in θ_p_ and Ω can change *R*_max_ (see eq 5).

Similarly, there exists a minimum droplet radius *R*_min_ below which jumping droplets cannot lift a nanoparticle from a superhydrophobic surface, even if the case lies in the orange-shaded region of the phase diagram. This lower condition for *R*_min_ stems from the enhanced viscous dissipation inside the droplets in the presence of a nanoparticle and is best demonstrated in Figure 4(b), where two droplets with *R* = 7.2 nm coalesce with a nanoparticle underneath but do not jump off the surface (Ω = 1.5, θ_p_ = 30°, *l* = 1.5 nm). Our previous study^10^ showed that, under identical conditions but in the absence of a nanoparticle, their coalescence would result in the merged droplet jumping off the superhydrophobic surface. A theoretical estimate of *R*_min_ is obtained by equating the translational kinetic energy of droplet jumping in the absence of a nanoparticle (∼*γR*^2^V*^2^, where V* ≡ *V*_*j*_/*V*_*i*_ is the scaled jumping speed) to the work done against *F*_adh_, i.e., the particle–surface work of adhesion *W*_adh_. Here, the expression for the jumping droplet’s translational kinetic energy is obtained by considering the coalescence of two inviscid droplets. This gives
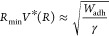
6Notably, while V* depends on *R*, *W*_adh_ depends on the nanoparticle size *l*. With the expression for V* obtained previously by curve fitting experimental data of jumping droplets,^29^eq 6 suggests that *R*_min_ is an increasing function of *l* (see the solid black line in Figure 4). Since 2*R*_min_ > *l* must be satisfied here as well for the jumping droplet to accommodate the dislodged nanoparticle (see the black dot-dashed line in the same figure), *R*_min_ would lie above both the solid and dot-dashed black lines in Figure 4(a), where both conditions for minimum droplets’ radii are satisfied. Therefore, theoretically, all droplet-size-related conditions for nanoparticle lifting removal are satisfied only in the orange-shaded region of Figure 4(a). Evidence of this limiting condition of *R*_min_ is shown using our MD simulations in Figure 4(a) for one particle size.

From the above observations, we conclude that decreasing nanoparticle–substrate adhesion (i.e., decreasing Ω) and/or increasing nanoparticle–condensate liquid interaction (i.e., decreasing θ_p_) will result in the upward shifting of the stiffness-limited maximum droplet size *R*_max_ and a downward shifting of *R*_min_, widening the orange-shaded region in Figure 4(a).

## Lateral Relocation

**Figure 5:**
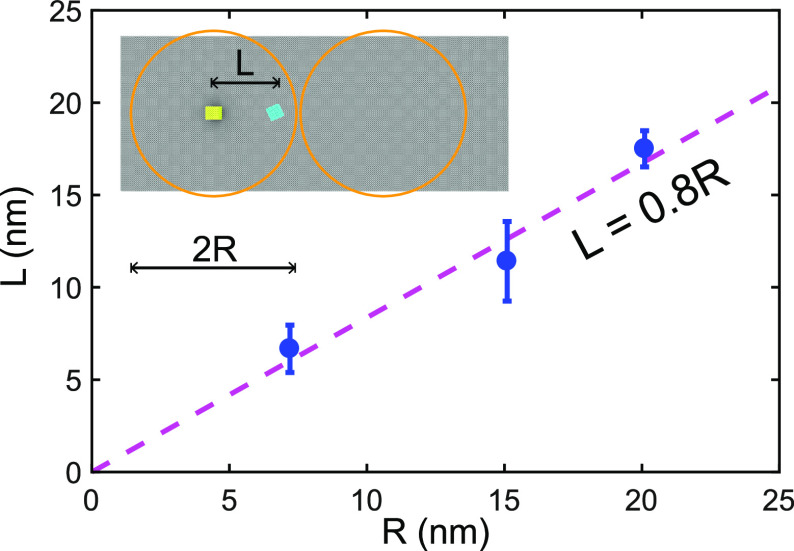
Lateral displacement (*L*) of nanoparticles on superhydrophobic surfaces following droplet coalescence and jumping. Inset: schematic showing the initial (yellow) and final (cyan) locations of a nanoparticle during coalescence of two droplets with radii *R*. The initial locations of the droplets are shown as two orange solid circles.

In addition to nanoparticle removal, our simulations reveal that, whenever coalescing droplets are not lifting particles on superhydrophobic surfaces, they can laterally displace them on the surface instead. Particle relocation is observed inside the red-shaded regions of Figure 2 (for any value of *R*) and Figure 4(a) (i.e., when either *R* > *R*_max_ or *R* < *R*_min_). In all cases, the nanoparticles are dragged on the surface by a distance comparable to the radius of the enclosing droplet, as shown in Figure 5. Such possibilities make condensate droplets a potential candidate for relocating individual nanoparticles on superhydrophobic surfaces by successive instances of droplet coalescence. A major advantage here is that the droplets autonomously condense on nanoparticles that act as nucleation sites. This new energy-efficient technique could help manipulate the location of nanoparticles with applications in single-molecule biophysics,^30^ biosensing,^31^ and detection of explosives,^32^ where existing energy-intensive methods lack control over individual particles.^33−35^ Here, the particle-enclosing droplet will grow as a result of coalescence with a neighbor as well as due to condensation.

In summary, we have precisely identified the conditions under which mechanisms of floating, lifting, or no removal of nanoparticles using droplets occur on different surfaces and have provided a new self-cleaning phase map that can guide future experiments. We reveal that floating removal can clear nanoparticles from any surface, provided the liquid/nanoparticle attractive forces overcome the solid/nanoparticle adhesive forces. For lifting removal, the underlying surface needs to be superhydrophobic, and we observe the droplet lifts the particle using a hot-air balloon configuration. An additional non-equilibrium force term added to the force balance now provides a reasonable condition that separates floating from lifting removal. The size of the condensate droplets has a crucial influence on these self-cleaning processes, especially on lifting removal. We additionally demonstrate for the first time that condensate-droplet-driven nanoparticle transport can be a powerful tool to laterally relocate individual nanoparticles on superhydrophobic surfaces, which can be exploited for local precision cleaning or precision assembly in future electronics and biosensors. Since condensation predominantly happens near nanoparticles acting as nucleation sites, this method does not involve the challenging task of exactly placing the droplets over the particles.

Future experiments can further validate the new theory by observing self-cleaning of superhydrophobic surfaces, where the size of the contaminants (*l*) is systematically varied. Having independent control over *l* will enable us to vary Ω. This procedure can be repeated using contaminants made up of various materials, which enables us to independently vary θ_p_. However, special care must be taken in the experiments to operate within the limits of droplet size-dependency of the self-cleaning outcome. Furthermore, the effects of surface texture, particle shape, and charge on floating, lifting, and lateral displacement mechanisms using coalescence-induced jumping droplets are not investigated here and therefore this work offers many opportunities for future research directions.

## Data Availability

The data and the script files for selected cases that support the findings of this study are available at https://doi.org/10.7488/ds/3853.

## Supporting Information Available

Simulation details as well as a comparison of the new theory with previous experiments (PDF)
Video showing different jumping-droplet-enabled surface self-cleaning mechanisms (MP4)
