# Supplementary material for: Contaminant Removal from Nature’s Self-Cleaning Surfaces
Source: Nano Lett. 2023 May 8;23(10):4234–41. doi: 10.1021/acs.nanolett.3c00257 (PMC10214492; doi:10.1021/acs.nanolett.3c00257)
Supplement: Supplementary file 1 — nl3c00257_si_001.pdf [file nl3c00257_si_001.pdf]

Supporting Information for the article

Contaminant Removal from Nature's Self-Cleaning Surfaces

submitted to *Nano Letters*

Sreehari Perumanath,[*,†] Rohit Pillai,[‡] and Matthew K Borg[‡]

†*Mathematics Institute, University of Warwick, Coventry CV4 7AL, UK*

‡*School of Engineering, University of Edinburgh, Edinburgh EH9 3FB, UK*

E-mail: sreehari.perumanath@warwick.ac.uk

1 Molecular Dynamics Simulations

MD simulations[1] of coalescence-induced jumping of water nanodroplets are performed to demonstrate the selectivity of the process in lifting or displacing nanoparticles from superhydrophobic surfaces. MD is a deterministic simulation tool in which the time evolution of a set of interacting molecules is carried out by integrating Newton's equations of motion. The net force on any atom is obtained from the negative gradient of the potential energy function due to all its neighbours. The simulations are performed using the LAMMPS software.[2]

We begin the simulation by constructing two droplets in a fully periodic domain, each with a specified number of molecules according to the intended size R. In our study, water is simulated using the mW mono-atomic water model.[3] The mW model approximates each water molecule as a single atom and the hydrogen bonding among these water molecules are described by the Stillinger-Weber potential with parameters calibrated to match the physical

properties of water. All other intermolecular interactions are modelled using the standard 12-6 shifted Lennard Jones (LJ) potential with ϵ and σ as the energy and length parameters.

Underneath one of the droplets, a cube-shaped rigid nanoparticle of length scale l is placed on a wall with FCC crystal structure and lattice parameter $a = 0.392$ nm. This shape enables us to quantify the force of adhesion between the nanoparticle and the wall that we use in validating our theoretical analysis (see below), although the theory is not limited by the particle shape. The thickness of the wall is chosen to be the fluid-wall LJ interaction cut-off distance, which is $r_c = 1.3$ nm. In the initial stages of equilibration, the two liquid droplets are placed on a *hydrophobic* wall for which the fluid-wall energy parameter is $\epsilon_{FW} \sim 0.1$ kcal/mol. Then, ϵ_{FW} is gradually reduced until the fluid-wall contact angle is increased to a value well above 150° at which point $\epsilon_{FW} \approx 0.01$ kcal/mol; this turns the hydrophobic surface into superhydrophobic. The full system is then equilibrated further for 5 ns. During the equilibration stage, a Berendsen thermostat is applied to the droplets and the time-integration is performed with a time-step size of 0.01 pico-seconds (ps). In all cases, the wall atoms are frozen to their initial lattice coordinates. After both droplets are equilibrated, the thermostat is removed and a sufficiently small impact speed (~ 1 m/s) is given to the droplet on the right side towards the other droplet, so that they will come together and coalesce.[4] We studied 3 independent realisations for any particular case.

In order to characterise the nature of the contaminant, we evaluate F_{adh} from a separate set of MD simulations consisting of only the wall and the contaminant. Here, after equilibration, the contaminant is displaced from the wall at small regular intervals, and the net force experienced by the contaminant from all wall atoms is recorded. Since the magnitude of this opposing force is dependent on the distance between the contaminant and the wall, we take F_{adh} as the maximum of this force. This procedure is repeated for various contaminant sizes as well as for different wall-contaminant interactions.

Similarly, the contaminant-liquid contact angle, θ_p, is determined in another set of MD simulations by spreading a liquid droplet on a slab of contaminant. Here, the thickness of

the slab is the cut-off distance used in the simulations. After steady-state is achieved and the droplet has acquired its equilibrium configuration, its density profile is determined. Circular bins are placed centred on a line passing through the droplet's centre-of-mass and normal to the contaminant slab. Afterwards, the equimolar points in the density profile, where the local density falls to the average of liquid and vapour bulk densities are obtained. A circle is then fit to these equimolar points of the droplet, and the tangent to this circle where it meets the contaminant slab gives θ_p.

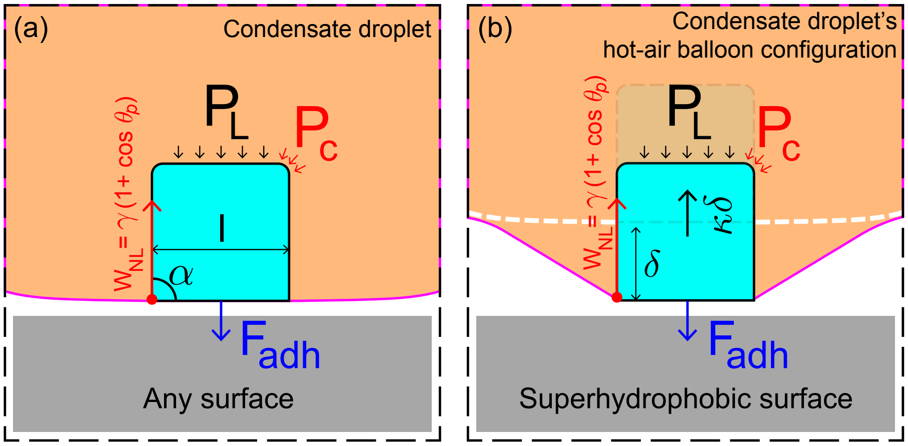

Figure S1: Free body diagram of the cube-shaped nanoparticle that is subjected to the force of adhesion from the surface F_{adh}, Laplace pressure and the dislodging capillary forces from the condensate liquid during (a) floating removal and (b) lifting removal.

2 Mechanisms of nanoparticle removal (additional information)

1. Floating removal. This type of nanoparticle removal happens when the attraction from the condensate liquid molecules on the particle is large enough to overcome its force of adhesion from the underlying surface. As shown in Fig. S1(a), once the size of the condensate droplet $2R > l$, the nanoparticle experiences various forces: (1) an upward pulling force (per unit length) of magnitude $W_{\text{NL}} = \gamma(1 + \cos\theta_p)$ that is tangential to the nanoparticle surface, owing to the attraction from the surrounding condensate molecules acting along the wetted

perimeter $P = 4l$ of the cube-shaped nanoparticle; (2) the Laplace pressure $P_L \sim \gamma/R$ acting downwards over an area $\sim l^2$ projected on to the underlying wall; (3) a force acting downwards due to pressure P_c arising because of the finite curvature of the nanoparticle surface that acts upon the curved area; and (4) the force of adhesion F_{adh} from the wall. Here, θ_p is the equilibrium contact angle between the condensate liquid and the nanoparticle, and γ is the liquid/vapour interfacial tension of the condensing liquid.

The Laplace pressure force $\sim \gamma l^2/R$ is smaller in magnitude by a factor l/R than other forces, which scale as γl, and is neglected. The total contribution from the pressure P_c integrated over the entire nanoparticle surface results in a force γP oriented downwards[5,6] along with F_{adh}. Consequently, the only dislodging force in this scenario is due to W_{NL} acting along P and the condition for floating removal becomes:

$$\gamma P(1 + \cos\theta_p) \geq F_{\text{adh}} + \gamma P. \tag{1}$$

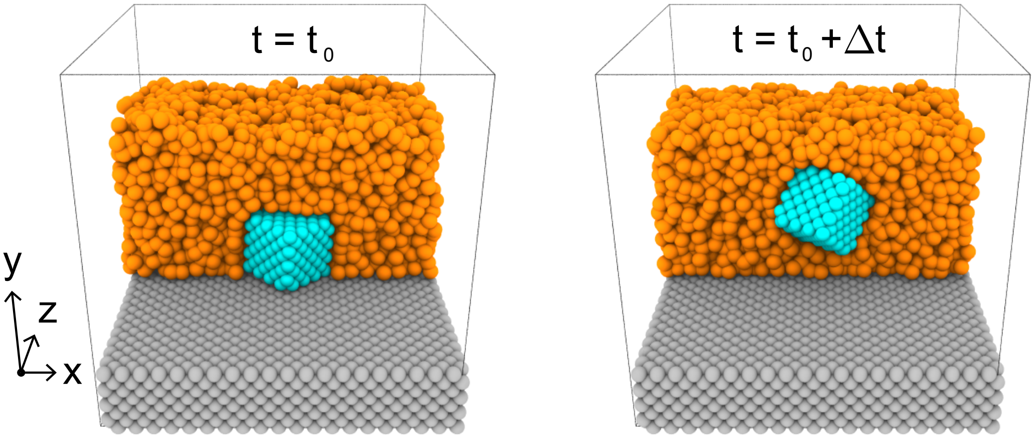

Figure S2: Floating removal observed in a simplified geometry, where the underlying wall was hydrophlic ($\theta_w \approx 40°$) instead of superhydrophobic ($\theta_w > 150°$). In the simulation snapshots, the frontal half of the water slabs are not shown.

In Fig. S2, we study floating removal in a simplified geometry, where a nanoparticle is placed under a liquid slab on a hydrophilic wall. The wall wettability is quantified by the

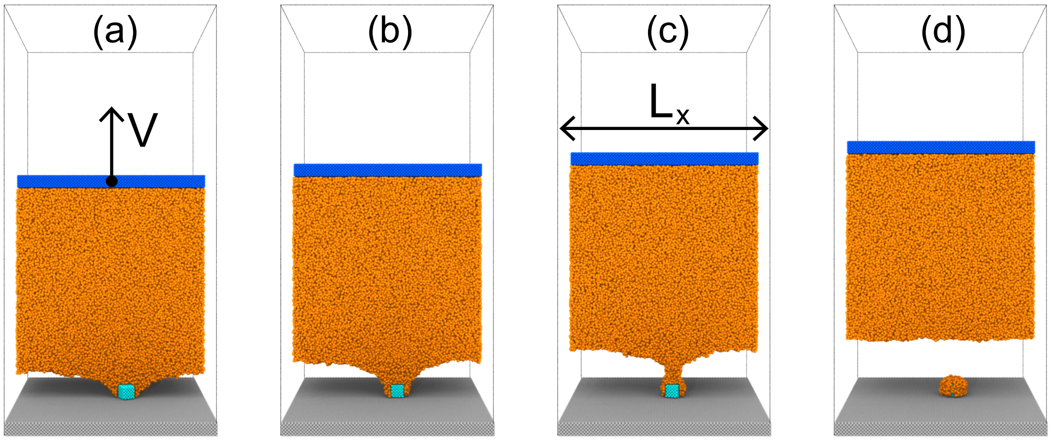

Figure S3: RP instability-induced liquid column break-up in a controlled set-up (l = 1.5 nm, $\Omega = 3.9$, $\theta_p = 0°$). The top wall moves upwards with a constant speed V. The liquid body leaves behind the nanoparticle in the process. In the figure, the frontal half of the liquid body and the moving top wall are not shown.

wetting contact angle θ_w between itself and the condensate liquid, and here, $\theta_w \approx 40°$. In these simulations, we observe floating removal in all cases where condition (1) is satisfied irrespective of θ_w.

2. Lifting removal. Here, an additional force is supplied by a component of the surface tension force that gains importance when the system reaches a hot-air balloon configuration (see Fig. 1(e) of the main Letter). The magnitude of this additional force will be $\kappa\delta$, where κ is the stiffness coefficient of the droplet surface acting as a linear spring for displacements $\delta \ll R$.

Analytical expressions exist for determining κ that generally depend on the relative size of the particle and the droplet, and the liquid surface tension. The stiffness coefficient when a particle is pulled from a droplet surface is given by:[7]

$$\kappa = \frac{4\pi\gamma}{2\ln(4R/l) - 1}. \tag{2}$$

For a particular value of l, equation (2) predicts that κ will decrease with increasing R. The above expression is used to derive equation (5) in the main Letter.

We demonstrate RP instability-induced liquid column break-up during droplet jumping by considering a simplified geometry that uses a liquid slab, as shown in Fig. S3. Here, we move the top wall with a constant speed that is comparable to the jumping speeds of water droplets lifting off from superhydrophobic surfaces. From our previous work, we estimated $V = 3$ m/s would be a reasonable value to use here.[4] In this case, the stiffness coefficient is given by:[8]

$$\kappa = \frac{2\pi\gamma}{\ln\left(2L_x/l\right)}, \tag{3}$$

where L_x is the length of the square simulation domain as shown in the figure. The fact that κ decreases with increasing L_x/l implies that the RP instability-induced break-up would be observed for this system even if we simulated it with a much larger domain size L_x (or jumping of a correspondingly large droplet).

3 Comparison of the new theory with previous experiments

Table S1: Estimated values of $\Omega \equiv F_{\mathrm{adh}}/\gamma P$ for previous experiments. In studies where researchers have not provided their own estimates of F_{adh} and θ_p, we have determined their values from other works, which are cited in the respective columns.

Experiments	Contaminant material	l (μm)	θ_p	F_{adh} (nN)	Ω
Geyer *et al.* (2020)[9]	Silica-coated glass<br>hydrophobized silica	50	60°<br>100°	2590	0.23
Abdelmagid *et al.* (2019)[10]	Environmental dust	1.2	6°[11]	30[12]	0.11
Heckenthaler *et al.* (2019)[13]	Silica	3	30°	900	1.32
Yilbas *et al.* (2018)[12]	Environmental dust	5.2	6°[11]	65	0.055
Watson *et al.* (2015)[14]	Silica	15	30°[13]	200[15]	0.06
Watson *et al.* (2014)[16]	Silica	20	30°[13]	200[15]	0.045
Wisdom *et al.* (2013)[17]	Glass,<br>PMMA	25	10°,<br>60°	2500[9]	0.45

Here, we determine Ω for various systems studied in previous experiments that have demonstrated self-cleaning either by jumping or by rolling of droplets on superhydrophobic

surfaces (see Table S1[†]). While some of the previous studies on self-cleaning surfaces have explicitly reported F_{adh} they estimated, others have not. In the latter cases, we determine F_{adh} from some other works which studied the adhesion of those particles on the same superhydrophobic surface. These values are given in the fifth column of Table S1, which are then used to evaluate Ω.

Furthermore, in experiments, unlike the cube-shaped nanoparticles that we have used here, the surface of the particle where the contact line is pinned may be inclined to the superhydrophobic surface by an angle α ($\alpha = 90°$ in Fig. 1(d) in the Letter). This results in an additional factor of $\sin\alpha$ in the denominator of the definition of Ω, as only this component of the surface tension will try to dislodge the particle from the surface. Another factor that we have omitted here is the effect of nanoparticle surface roughness, which results in the actual wetted perimeter nP ($n > 1$) to be larger than the nominal wetted perimeter $P \approx \pi l$. While evaluating Ω, we have assumed that the effect of these two factors balance, i.e. $\sin\alpha \sim 1/n$. The results are plotted in Fig. 2(b) of the Letter and we observe good agreement with our theory, which justify the assumptions made.

References

(1) Allen, M. P.; Tildesley, D. J. *Computer Simulation of Liquids*, 2nd ed.; Oxford University Press: New York, 2017.

(2) Plimpton, S. Fast Parallel Algorithms for Short – Range Molecular Dynamics. *J. Comput. Phys.* **1995**, *117*, 1–19.

(3) Molinero, V.; Moore, E. B. Water Modeled As an Intermediate Element between Carbon and Silicon. *J. Phys. Chem. B* **2009**, *113*, 4008–4016.

[†]Note that Geyer *et al.*[9] used the so-called JKR theory to estimate F_{adh} irrespective of particle size l. However, the validity of this theory is questionable when l is comparable with the roughness scale of the underlying surface. In other words, the actual value of Ω will be larger than 0.23.

(4) Perumanath, S.; Borg, M. K.; Sprittles, J. E.; Enright, R. Molecular physics of jumping nanodroplets. *Nanoscale* **2020**, *12*, 20631–20637.

(5) Marchand, A.; Weijs, J. H.; Snoeijer, J. H.; Andreotti, B. Why is surface tension a force parallel to the interface? *Am. J. Phys.* **2011**, *79*, 999–1008.

(6) Das, S.; Marchand, A.; Andreotti, B.; Snoeijer, J. H. Elastic deformation due to tangential capillary forces. *Phys. Fluids* **2011**, *23*, 072006.

(7) Lishchuk, S. V.; Ettelaie, R. Detachment Force of Particles with Pinning of Contact Line from Fluid Bubbles/Droplets. *Langmuir* **2016**, *32*, 13040–13045.

(8) Tang, Y.; Cheng, S. Capillary forces on a small particle at a liquid-vapor interface: Theory and simulation. *Phys. Rev. E* **2018**, *98*, 1–15.

(9) Geyer, F.; D'Acunzi, M.; Sharifi-Aghili, A.; Saal, A.; Gao, N.; Kaltbeitzel, A.; Sloot, T.-F.; Berger, R.; Butt, H.-J.; Vollmer, D. When and how self-cleaning of superhydrophobic surfaces works. *Sci. Adv.* **2020**, *6*, eaaw9727.

(10) Abdelmagid, G.; Yilbas, B. S.; Al-Sharafi, A.; Al-Qahtani, H.; Al-Aqeeli, N. Water droplet on inclined dusty hydrophobic surface: influence of droplet volume on environmental dust particles removal. *RSC Adv.* **2019**, *9*, 3582–3596.

(11) Wu, W.; Giese, R. F.; Van Oss, C. J. Change in surface properties of solids caused by grinding. *Powder Technol.* **1996**, *89*, 129–132.

(12) Yilbas, B. S.; Hassan, G.; Al-Sharafi, A.; Ali, H.; Al-Aqeeli, N.; Al-Sarkhi, A. Water Droplet Dynamics on a Hydrophobic Surface in Relation to the Self-Cleaning of Environmental Dust. *Sci. Rep.* **2018**, *8*, 1–19.

(13) Heckenthaler, T.; Sadhujan, S.; Morgenstern, Y.; Natarajan, P.; Bashouti, M.; Kaufman, Y. Self-Cleaning Mechanism: Why Nanotexture and Hydrophobicity Matter. *Langmuir* **2019**, *35*, 15526–15534.

(14) Watson, G. S.; Schwarzkopf, L.; Cribb, B. W.; Myhra, S.; Gellender, M.; Watson, J. A. Removal mechanisms of dew via self-propulsion off the gecko skin. *J. R. Soc. Interface* **2015**, *12*, 20141396.

(15) Hu, H. M.; Watson, J. A.; Cribb, B. W.; Watson, G. S. Fouling of nanostructured insect cuticle: Adhesion of natural and artificial contaminants. *Biofouling* **2011**, *27*, 1125–1137.

(16) Watson, G. S.; Gellender, M.; Watson, J. A. Self-propulsion of dew drops on lotus leaves: a potential mechanism for self cleaning. *Biofouling* **2014**, *30*, 427–434.

(17) Wisdom, K. M.; Watson, J. A.; Qu, X.; Liu, F.; Watson, G. S.; Chen, C.-H. Self-cleaning of superhydrophobic surfaces by self-propelled jumping condensate. *Proc. Natl. Acad. Sci. U. S. A.* **2013**, *110*, 7992–7.
